# Doxofylline ameliorates liver fibrosis by regulating the ferroptosis signaling pathway

**DOI:** 10.3389/fphar.2023.1135366

**Published:** 2023-03-17

**Authors:** Lenan Xu, Meiling Zhang, Junzhi Pan, Xiangwei Xu, Yawen Zhang, Xue Han, Lina Yin, Lingfeng Chen, Juan Ren, Jie Yu, Yanmei Zhang, Guang Liang, Yi Zhang

**Affiliations:** ^1^ Affiliated Yongkang First People’s Hospital, School of Pharmacy, Hangzhou Medical College, Hangzhou, Zhejiang, China; ^2^ School of Pharmaceutical Sciences, Hangzhou Medical College, Hangzhou, Zhejiang, China; ^3^ Zhejiang Provincial Key Laboratory of Laboratory Animals and Safety Research, Hangzhou Medical College, Hangzhou, Zhejiang, China; ^4^ School of Laboratory Medicine and Bioengineering, Hangzhou Medical College, Hangzhou, Zhejiang, China; ^5^ Chemical Biology Research Center, School of Pharmaceutical Sciences, Wenzhou Medical University, Wenzhou, Zhejiang, China

**Keywords:** liver fibrosis, doxofylline, hepatic stellate cells, ferroptosis, deferoxamine (DFO)

## Abstract

Liver fibrosis, a compensatory repair response to chronic liver injury, is caused by various pathogenic factors, and hepatic stellate cell (HSC) activation and phenotypic transformation are regarded as key events in its progression. Ferroptosis, a novel form of programmed cell death, is also closely related to different pathological processes, including those associated with liver diseases. Here, we investigated the effect of doxofylline (DOX), a xanthine derivative with potent anti-inflammatory activity, on liver fibrosis as well as the associated mechanism. Our results indicated that in mice with CCl_4_-induced liver fibrosis, DOX attenuated hepatocellular injury and the levels of liver fibrosis indicators, inhibited the TGF-β/Smad signaling pathway, and significantly downregulated the expression of HSC activation markers, both *in vitro* and *in vivo*. Furthermore, inducing ferroptosis in activated HSCs was found to be critical for its anti-liver fibrosis effect. More importantly, ferroptosis inhibition using the specific inhibitor, deferoxamine (DFO) not only abolished DOX-induced ferroptosis, but also led to resistance to the anti-liver fibrosis effect of DOX in HSCs. In summary, our results showed an association between the protective effect of DOX against liver fibrosis and HSC ferroptosis. Thus, DOX may be a promising anti-hepatic fibrosis agent.

## Introduction

Liver fibrosis, which is characterized by a disease course that is long and reversible, is a repair response to chronic liver injury caused by various factors. Without active and effective intervention, it can progress to liver cirrhosis or even liver cancer (Lee et al., 2015). Therefore, developing effective liver fibrosis prevention and reversal strategies has become a major challenge worldwide. Current mainstream studies suggest that hepatic stellate cell (HSC) activation is a key event in the development of liver fibrosis. With increasing interest in developing antifibrotic therapies, there is a need for cell lines that preserve the *in vivo* phenotype of human HSCs to elucidate pathways of human hepatic fibrosis. Therefore, LX-2 was established which characterised human HSC cell lines. LX-2 was generated by spontaneous immortalisation in low serum conditions. LX-2 express α smooth muscle actin, vimentin, and glial fibrillary acid protein, as visualised by immunocytochemistry. Similar to primary HSCs, LX-2 express key receptors regulating liver fibrosis, including platelet derived growth factor receptor β (PDGF-R), obese receptor long form (Ob-RL), and discoidin domain receptor 2 (DDR2), and also proteins involved in matrix remodelling; matrix metalloproteinase (MMP)-2, tissue inhibitor of matrix metalloproteinase (TIMP)-2, and MT1-MMP, as determined by western analyses. LX-2 had a retinoid phenotype typical of stellate cells. Microarray analyses showed strong similarity in gene expression between primary HSCs and either LX-2 (98.7%), with expression of multiple neuronal genes (Xu et al., 2005). Activited HSC affects the normal functioning of hepatocytes and Kupffer cells, further aggravating the pathological state of the liver (Tacke and Trautwein, 2015). Therefore, inhibiting the activation and proliferation of HSCs and inducing their death is a plausible treatment strategy for liver fibrosis.

Ferroptosis is a form of programmed cell death caused by iron-dependent oxidative damage. Unlike other forms of programmed cell death, ferroptosis is characterized by the excessive accumulation of lipid reactive oxygen species (ROS), mitochondrial wrinkling, and increased mitochondrial membrane density (Dixon et al., 2012; Li et al., 2020). Intracellular iron level is mainly regulated by transferrin receptor which accounts for transporting extracellular iron-transferrin complex into cells *via* clathrin-mediated endocytosis, ferritin composed of light chain and heavy chain and responsible for storing iron, and ferroportin in charge of iron exportation. The increased iron disrupts membrane integrity by peroxidizing polyunsaturated fatty acid (PUFA) chains of membrane phospholipids *via* two pathways. One is to serve as a cofactor for nonheme iron-containing lipoxygenase to enzymatically catalyze PUFA peroxidation. The other is to react with H_2_O_2_ *via* Fenton reaction to generate toxic hydroxyl radicals, which have potent capacity to peroxidize PUFA (Ooko et al., 2015; Chen et al., 2021). In recent years, the role of ferroptosis in the development of liver fibrosis has attracted increased attention and has become a research hotspot in the field of liver fibrosis. Notably, several studies have shown that large amounts of Fe^2+^ are stored in HSCs and that ferroptosis can affect the development of liver fibrosis by regulating the Fe^2+^ content of HSCs as well as the degree of lipid peroxidation. Therefore, targeting HSC ferroptosis could be a novel strategy to treat liver fibrosis (Anderson and Shah, 2013).

**FIGURE 1:**
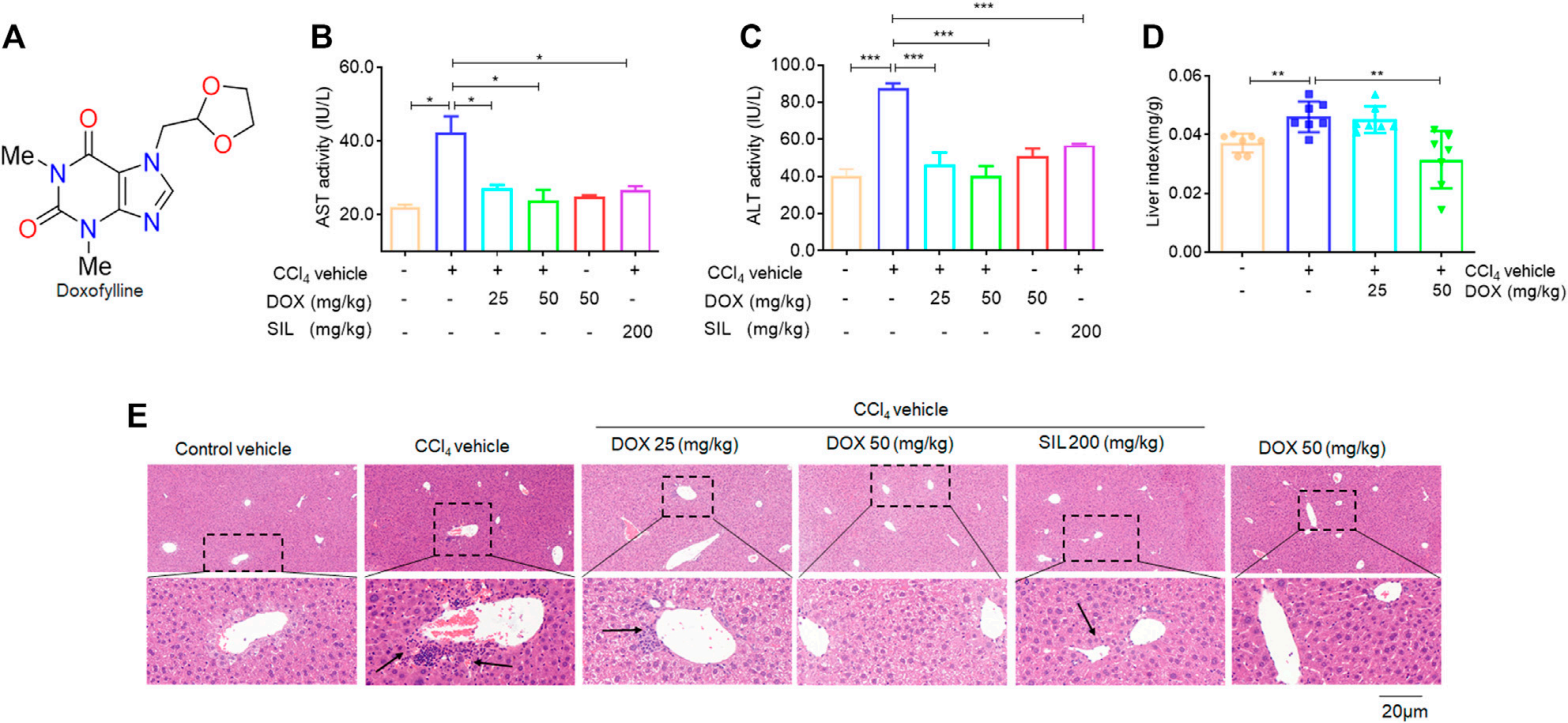
Effects of DOX serum ALT/AST activity and liver H&E staining. **(A)** The chemical structures of DOX. **(B)** AST activity. **(C)** ALT activity. Data were expressed as means ± SEM (*n* = 3). **(D)** Liver index (*n* = 7). **(E)** Liver H&E staining. Typical images were chosen from each experimental group. (original magnification ×400, upper images; partial enlarged pictures, down images). *p < 0.05; ***p < 0.001.

Additionally, doxofylline (DOX, Figure 1A), a xanthine derivative with a long-acting bronchodilator effect (Page, 2010), is regarded as a substitute for theophylline owing to its higher efficacy and fewer side effects (Matera et al., 2017). DOX has also been reported to be a non-selective phosphodiesterase (PDE) inhibitor with good anti-inflammatory activity. However, its effects on liver fibrosis remain unclear. Therefore, in this study, we attempted to clarify its effect on ferroptosis and further elucidate the mechanism underlying its action in liver fibrosis.

## Materials and methods

### Chemical compounds and reagents

Kits for detecting serum alanine (ALT)/aspartate aminotransferase (AST) activity, liver hydroxyproline content, and glutathione (GSH) from LX-2 cells were purchased from Jiancheng Bioengineering Institute (Nanjing, China). The Phen Green SK diacetate (PGSK) kit was purchased from Glpbio (Montclair, CA, United States), cell counting Kit-8 (CCK-8) was purchased from APE×BIO Technology LLC (Houston, TX, United States), the 2′,7′-dichlorodihydrofluorescein diacetate (DCFH-DA) assay kit was purchased from Solarbio (Beijing, China) and MitoLite™ Red FX600 Kit was purchased from AAT Bioquest, Inc. (Sunnyvale, CA, United States). 2.5% glutaraldehyde solution was purchased from Solarbio (Beijing, China). Further, antibodies against α-smooth muscle actin (α-SMA), vimentin (VIM), desmin (DES), SMAD family member 2/3 (Smad 2/3), p-Smad 2/3, transforming growth factor-beta (TGF-β), glutathione peroxidase 4 (GPX4), solute carrier family-7 member-11 (SLC7A11), solute carrier family 40 member 1 (SLC40A1), transferrin, transferrin receptor and glyceraldehyde-3-phosphate dehydrogenase (GAPDH) were purchased from Cell Signaling Technology (Danvers, MA, United States). Furthermore, the FDA staining kit was purchased from AAT Bioquest (Sunnyvale, CA, United States), FerroOrange was purchased from Goryo Chemical Inc. (Hokkaido, Japan), and deferoxamine (DFO) was purchased from Sigma-Aldrich (St. Louis, MO, United States). Peroxidase-conjugated or fluorescein isothiocyanate (FITC)-conjugated antibodies were purchased from Jackson ImmunoResearch (West Grove, PA, United States), TRIzol reagent was purchased from Invitrogen Life Technology (Carlsbad, CA, United States), the PrimeScript Master Mix and SYBR Premix Ex Taq were purchased from Yeasen Biotechnology Co., Ltd. (Shanghai, China). Doxofylline (DOX) and silymarin (SIL) were purchased from Yuanye Bio-Technology Co., Ltd. (Shanghai, China).

### Experimental animals

C57BL/6 mice (20 ± 2 g) were purchased from the Experimental Animal Center of Hangzhou Medical College (Hangzhou, China). The animals were maintained under controlled temperature (22°C ± 1°C), humidity (50%), and light (12-h light/12-h dark cycle) conditions. All the animals received humane care according to the institutional animal care guidelines of the Experimental Animal Ethical Committee of Hangzhou Medical College. The mice were randomly divided into six groups, namely, the: 1) vehicle control (*n* = 7), 2) CCl_4_ model (*n* = 7), 3) CCl_4_+DOX (25 mg/kg) (*n* = 7), 4) CCl_4_+DOX (50 mg/kg) (*n* = 7), 5) DOX (50 mg/kg) (*n* = 7), and 6) CCl_4_+silymarin (SIL) (0.2 g/kg) (*n* = 5) groups. Thereafter, that mice were treated for 2 weeks with CCl_4_ (intraperitoneal injection, mixed with olive oil in the ratio 1:3, 2 mL/kg twice per week) and then for additional 4 weeks with both CCl_4_ (twice per week), DOX or SIL (intragastric administration, every day). After the treatment period, the mice were sacrificed, and their plasma and liver tissue samples were collected.

### Analysis of serum ALT/AST activities

Serum ALT and AST (Nanjing Jiancheng Bioengineering Institute) activities were assessed according to the manufacturer’s instructions.

### Liver histological evaluation

Liver tissue samples were fixed in 4% paraformaldehyde for 24 h, sectioned (4 μm) and stained with haematoxylin-eosin (H&E) for the histological observation of liver injury, Staining with masson’s trichrome and sirius red were also performed to observe collagen deposition in the liver of the mice.

### Measurement of liver hydroxyproline content

Liver hydroxyproline content was determined using the alkaline hydrolysis method in accordance with the manufacturer’s instruction (Nanjing Jiancheng Bioengineering Institute, Nanjing, China).

### Immunofluorescence staining with α-SMA

The liver sections were incubated overnight with α-SMA antibody in a humidified chamber at 4°C. Thereafter, the sections were incubated again with FITC-conjugated secondary antibody for 1 h. Finally, images were captured using an inverted IX81 microscope (Olympus, Tokyo, Japan).

### Immunohistochemical staining

Liver sections were blocked with 3% hydrogen peroxide and 3% bovine serum albumin (BSA) for 1 h. After rinsing once with PBS (phosphate buffer saline), diluted vimentin antibody was added to the sections followed by incubation overnight at 4°C. Next, goat anti-rabbit secondary antibody was added dropwise and the mixture was incubated at room temperature for 1 h. After washing with PBS, the tissue sections were developed with 3,3-Diaminobenzidine tetrahydrochloride (DAB) chromogenic solution and the nuclei were counterstained with hematoxylin. This was followed by thorough washing, after which the sections were dehydrated using 75% and 85% alcohol for 6 min and then washed with absolute ethanol and n-butanol, respectively, until they became transparent. Next, the tissue slices were then mounted on slides and their pathological characteristics were observed under a light microscope.

### Cell culture

LX-2 cells were cultured in dulbecco’s modified eagle medium (DMEM) supplemented with 10% [v/v] fetal bovine serum (FBS), 2 mM glutamine, 100 U/mL penicillin, and 100 mg/mL streptomycin. Heparg cells were cultured in RPMI1640 supplemented with 10% [v/v] fetal bovine serum, 2 mM glutamine, 100 U/mL penicillin and 100 mg/mL streptomycin.

### Cell viability assay

For assay of viability, LX-2 cells and heparg cells was seeded onto 96-well plates with a density of 5 × 10^3^ cells per well and treated by DOX (25, 50, 100, and 250 μM) for 48 h. Viability of cells in the 96-well plates were assayed using a CCK-8 kit (APE×BIO Technology, United States). The CCK-8 was incubated with cells at 37°C for 1 h. Then the 96-well plates was measured using a multifunctional enzyme labeler (Molecular devices, CA) at 405 nm.

### Cell death

Cell death was assessed using a FDA staining kit (AAT Bioquest Sunnyvale, CA). LX-2 cells were seeded onto 6-well plates with a density of 1 × 10^5^ cells per well and treated by DOX (10, 25 μM) for 48 h. Briefly, cells were stained in 10 μM FDA (Beyotime, China) in PBS for 1 h at 37°C. Finally, the samples were washed with PBS and imaged immediately. The samples were observed with a fluorescence inverted microscope (EVOS M7000, United States).

### Transmission electron microscopy

LX-2 cells were seeded onto 6-well plates with a density of 1 × 10^5^ cells per well and treated by DOX (25 μM) for 48 h. The cells were harvested by centrifugation at 3,000 rpm for 5 min. Then the cells were fixed by adding 2.5% glutaraldehyde solution. The samples were observed by the bio-TEM (Hitachi H-7000, Japan).

### GSH measurement

To measure total GSH levels, LX-2 cells was seeded onto 6-well plates with a density of 1 × 10^5^ cells per well and treated by DOX (10, 25 μM) for 48 h. The samples were determined using a glutathione assay kit (Nanjing Jiancheng Bioengineering Institute, China) following a standard protocol.

### ROS measurements

To measure ROS levels, LX-2 cells was seeded onto 6-well plates with a density of 1 × 10^5^ cells per well and treated by DOX (10, 25 μM) for 48 h. The cells were stained in 10 μM 2,7-Dichlorodi-hydrofluorescein diacetate, DCFH-DA (Solarbio, China) in DMEM for 1 h at 37°C. Next the samples were washed with PBS and imaged. The samples were observed with a fluorescence inverted microscope (EVOS M7000, United States).

### Iron level determination

Iron levels were measured as described below. LX-2 cells were seeded onto 6-well plates with a density of 1 × 10^5^ cells per well and treated by DOX (10, 25 μM) for 48 h. The cells were stained in 10 μM Phen Green SK (Glpbio, United States) in PBS for 1 h at 37°C. Finally, the samples were imaged immediately. LX-2 cells were seeded onto 6-well plates with a density of 1 × 10^5^ cells per well and treated by DOX (10, 25 μM) for 48 h. The cells were stained in 1 mM Ferro orange (Dojingo, Japan) in PBS for 1 h at 37°C. Next nuclei of the LX-2 cells were incubated with DAPI (Biosharp, China) for 30 min. Finally, the constructs were imaged immediately. All samples as described above were observed with a fluorescence inverted microscope (EVOS M7000, United States).

### Mitochondrion detection

Mitochondria were stained using MitoLite™ Red FX600 Kit (AAT Bioquest, United States). LX-2 cells were seeded onto 6-well plates with a density of 1 × 10^5^ cells per well and treated by DOX (10, 25 μM) for 48 h. Next cells were stained in the buffer containing a 1:500 dilution of Mitolite™ Red FX600 for 1 h at 37°C. Then nuclei of the LX-2 cells were incubated with DAPI (Biosharp, China) for 30 min. Finally, the samples were washed with PBS three times again. The samples were observed with a fluorescence inverted microscope (EVOS M7000, United States).

### Real-time PCR analysis

Total ribonucleic acid (RNA) was extracted from liver tissue samples and LX-2 cells and heparg cells using TRIzol reagent. Next, complementary deoxyribonucleic acid (cDNA) was synthesized using the PrimeScript RT Master Mix kit, and real-time PCR was performed using the SYBR green premix according to the manufacturer’s instructions. The relative expression levels of target genes were then normalized to actin, analyzed using the delta-delta-C_T_ method, and expressed as ratios relative to the vehicle control group. The primers used are listed in Supplementary Table S1.

### Western blot analysis

Liver and cell protein samples from mice in the different groups were isolated using a lysis buffer. This was followed by centrifugation at 3,000 g for 20 min at 4°C, after which supernatant samples were collected. Next, the protein concentration in each sample was then determined and normalized. Thereafter, the protein samples were separated *via* sodium dodecyl sulfate-polyacrylamide gel electrophoresis and transferred onto polyvinylidene fluoride membranes, which were then incubated with primary and secondary antibodies followed by the visualization of the proteins in the cell membranes using a chemiluminescence kit.

### Statistical analysis

Data are expressed as mean ± standard error of the mean (SEM). Further, significant differences were determined by performing one-way analysis of variance (ANOVA) with least significant difference (LSD) *post hoc* tests, and statistical significance was set at *p <* 0.05.

## Results

### DOX attenuated CCl_4_-induced liver injury in mice

In this study, we investigated whether therapeutic DOX administration could improve fibrotic liver damage. A classic CCl_4_-induced hepatic fibrosis mouse model was established, followed by DOX treatment. The therapeutic effect of DOX was then investigated by detecting changes in liver injury indices. From Figures 1B, C, it is evident that DOX treatment (25 and 50 mg/kg) significantly reduced serum AST/ALT activity, compared with the CCl_4_ model group. Reportedly, the liver index (liver weight/body weight ratio) has been found to be positively correlated with the degree of liver damage (Wu et al., 2015). Similarly, our results indicated that mice in the model group demonstrated significantly higher liver weight/body weight ratios than their counterparts in the normal group, and DOX treatment significantly lowered this index relative to its value for the model group (Figure 1D). Moreover, H&E staining of pathological liver sections showed that DOX ameliorated CCl_4_-induced liver injury in mice, reversing inflammatory cell infiltration and hepatocyte necrosis (Figure 1E). SIL is a well-known hepato-protective drug (Abd Elmaaboud et al., 2021). The results of serum ALT/AST activity and liver histological evaluation also indicated that it attenuates CCl_4_-induced liver injury (Figures 1B, C, E). Additionally, the DOX-only treatment (50 mg/kg) exert any significant effect on serum ALT/AST level and liver histomorphology compared with these characteristics for mice in the control group (Figures 1B, C, E). Therefore, DOX significantly improved the pharmacological effects of CCl_4_-induced liver injury in mice.

### DOX attenuated CCl_4_-induced liver fibrosis in mice

**FIGURE 2:**
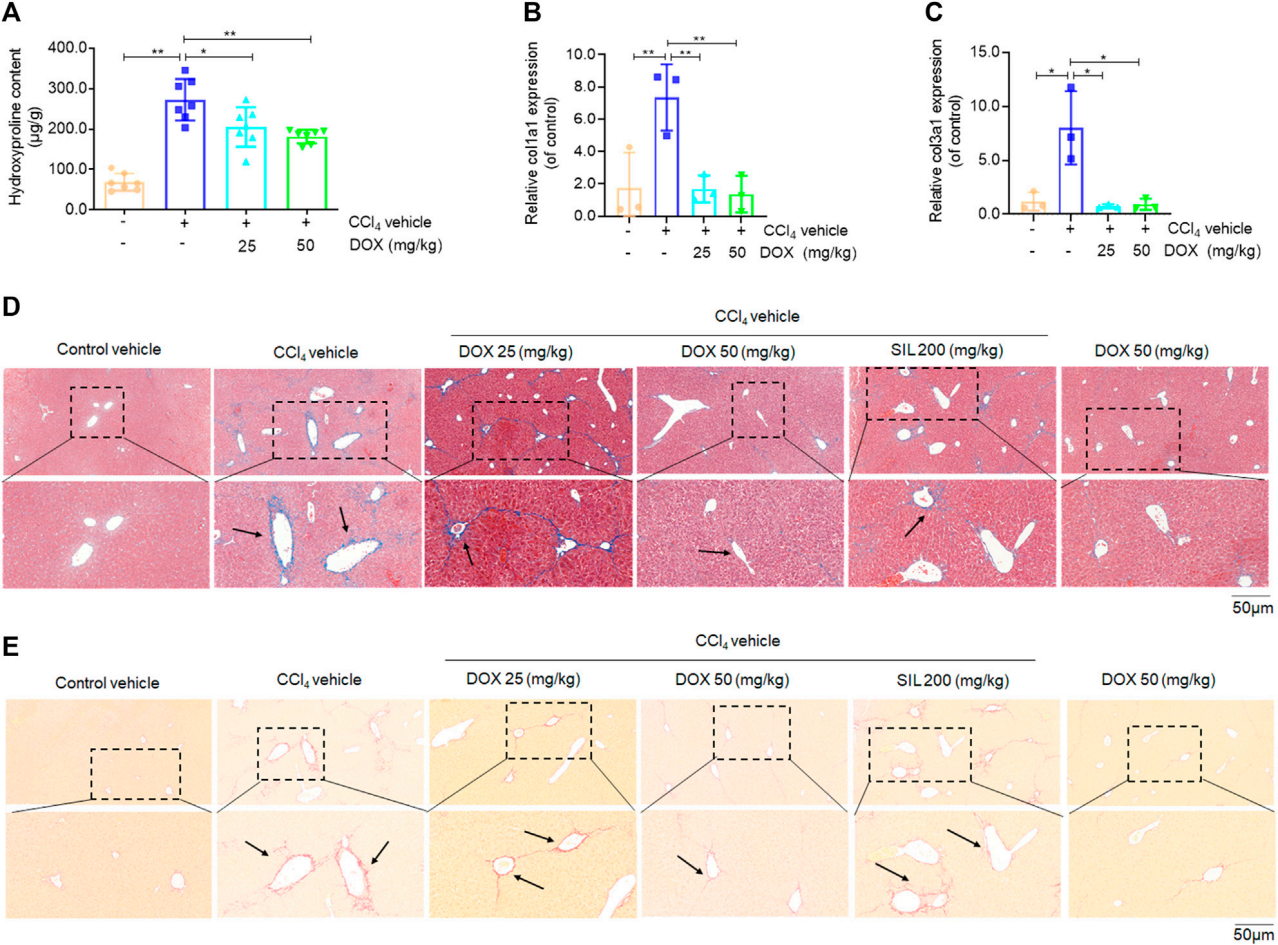
Effects of DOX on liver hydroxyproline content, serum markers of fibrosis and liver collagen expression. **(A)** Liver hydroxyproline content (*n* = 7). **(B,C)** Liver Col1a1 (Collagen, type I, α1) and Col3a1 (Collagen, type III, α1) (*n* = 3). **(D)** Liver masson’s trichrome staining. Typical images were chosen from each experimental group. Black arrows indicate collagen deposition. (original magnification ×200, upper images; partial enlarged pictures, down images). **(E)** Liver sirius red staining. Typical images were chosen from each experimental group. Black arrows indicate collagen deposition. (original magnification ×200, upper images; partial enlarged pictures, down images). Data were expressed as means ± SEM. *p < 0.05; **p < 0.01.

Next, we determined how DOX alleviated pathological changes in CCl_4_-induced hepatic fibrosis in mice. First, we assessed the level of hydroxyproline, which is considered as a gold standard marker for liver fibrosis (Wu et al., 2015). The results thus obtained showed that hydroxyproline was significantly elevated in liver tissue samples from mice in the CCl_4_ model group, while the administration of DOX (25 and 50 mg/kg) significantly alleviated this phenomenon (Figure 2A). Additionally, DOX administration resulted in a significant decrease in the upregulated liver Col1a1 and Col3a1 mRNA expression levels induced by CCl_4_ (Figures 2B, C). Our results also indicated that the pathogenesis of liver fibrosis involved the excessive accumulation of ECM, which has as main component, collagen. In this study, we used two staining methods (Masson’s trichrome and Sirius red staining) to investigate the effect of DOX on collagen deposition. The two staining results showed increased collagen deposition in samples from mice in the CCl_4_-induced liver fibrosis group, while this effect was completely normalized by DOX treatment (Figures 2D, E). In contrast, the DOX-only treatment of mice without liver fibrosis had no significant effect on collagen deposition in mouse liver tissue (Figures 2D, E).

### DOX inhibited HSCs activation in CCl_4_-treated mice

**FIGURE 3:**
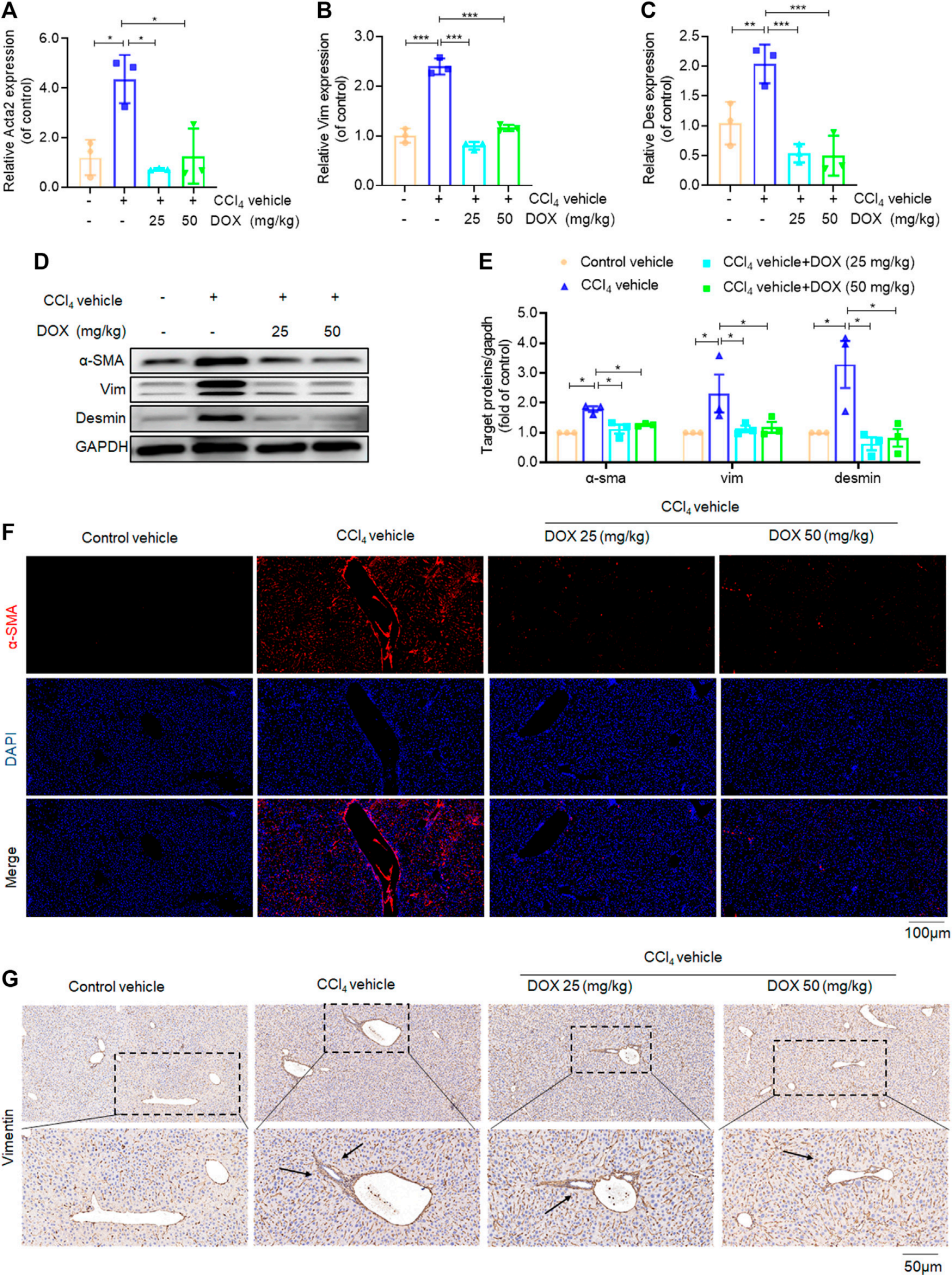
Effects of DOX on HSCs activation *in vivo*. **(A–C)** Liver Acta2 (α-SMA), Vim (Vimentin), Des (Desmin) mRNA expression (*n* = 3). **(D,B)** The expression of α-SMA, vim and desmin proteins in CCl_4_-induced mice liver tissues treated with DOX (25, 50 mg/kg) was detected by Western blot, and GAPDH was used as a loading control. **(E)** The quantitative result of α-SMA, vim and desmin. The results represent three independent experiments. **(F)** Liver α-SMA immunofluorescence staining (original magnification×100). **(G)** Liver Vim immunohistochemical staining (original magnification×100). Typical images are chosen from each experimental group. Black arrows indicate positive staining. Data were expressed as means ± SEM. *p < 0.05; **p < 0.01; ***p < 0.001.

Activated HSCs constitute the main source of ECM, hence play a major role in hepatic fibrosis. In this study, we investigated the inhibitory effects of DOX on HSC activation *in vivo*, based the mRNA levels of α-SMA, vimentin, and desmin, which are markers of activated HSCs (Puche et al., 2013; Greenhalgh et al., 2015). Our results in this regard showed that DOX treatment (25 and 50 mg/kg) significantly downregulated the mRNA and protein expression levels of hepatic α-SMA, vimentin, and desmin, compared with their levels for the CCl_4_ group (Figures 3A–E). Moreover, hepatic α-SMA immunofluorescence staining showed that DOX treatment resulted in an obvious decrease in the number of α-SMA-positive cells in CCl_4_-treated mice (Figure 3F). Similarly, hepatic vimentin immunohistochemical staining showed that DOX treatment resulted in a significantly reduced number of vimentin-positive cells in CCl_4_-treated mice (Figure 3G).

### DOX suppressed HSCs activation by inhibiting TGF-β/smad signaling *in vivo*

**FIGURE 4:**
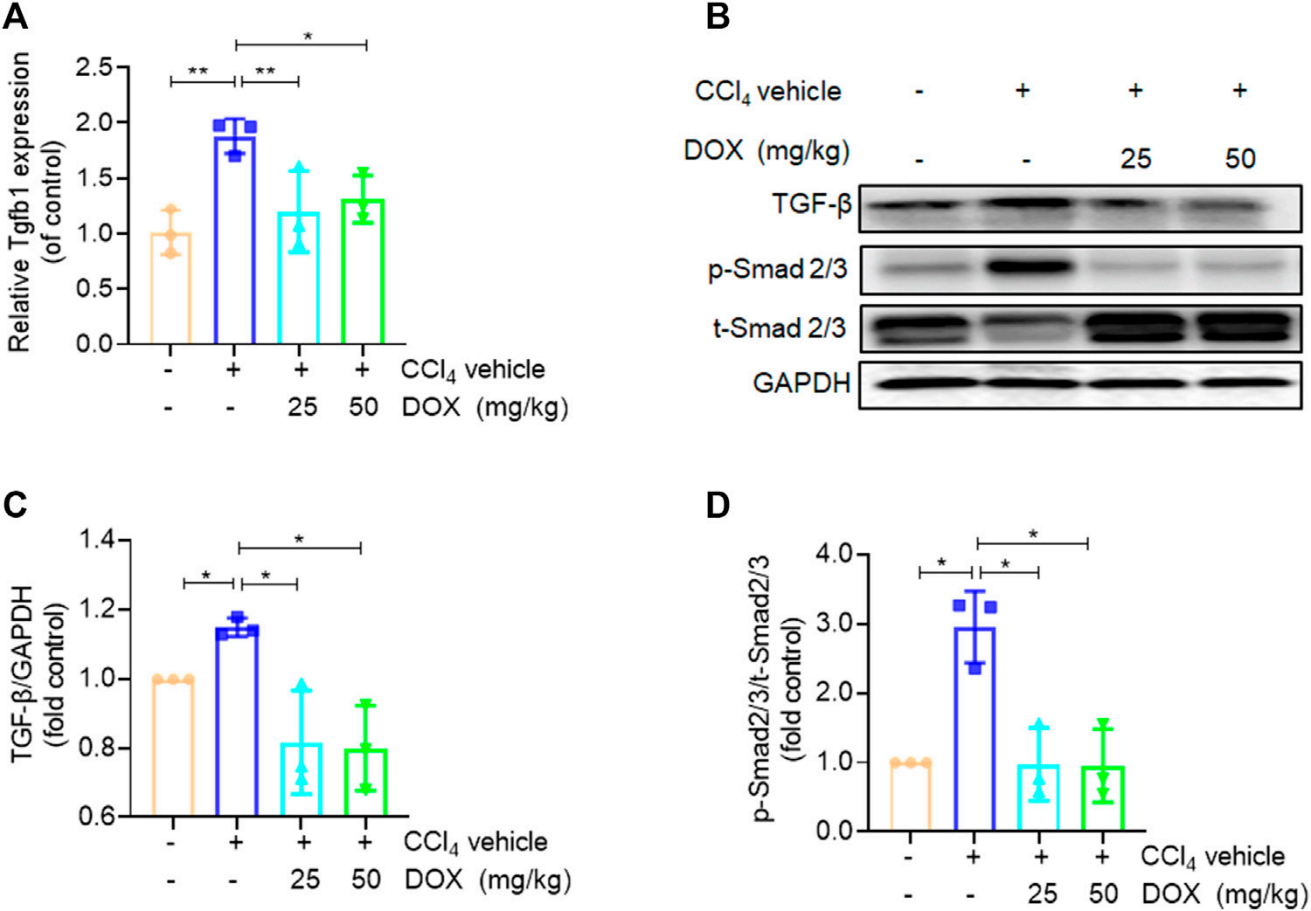
Effects of DOX TGF-β/Smad signalling *in vivo*. **(A)** Liver Tgfb1 (TGF-β), mRNA expression (*n* = 3). **(B)** The expression of TGF-β, p-Smad2/3 and t-Smad 2/3 proteins in CCl_4_-induced mice liver tissues treated with DOX (25 mg/kg, 50 mg/kg) was detected by Western blot, and GAPDH was used as a loading control. The results represent three independent experiments. **(C)** The quantitative result of TGF-β. The results represent three independent experiments. **(D)** The quantitative result of p-Smad2/3. The results represent three independent experiments. Data were expressed as means ± SEM. *p < 0.05; **p < 0.01.

TGF-β, which shows association with all key links in the pathogenesis of liver fibrosis, is one of the most potent pro-fibrotic cytokines. This implies that the TGF-β/Smad signaling pathway plays an extremely important role in the activation of HSCs (Yang et al., 2003). In this study, we further used real-time PCR and western blot analysis to investigate the effect of DOX on the key proteins of this signaling pathway. The results of real-time PCR showed that DOX treatment decreased TGF-β mRNA expression in mice (25 and 50 mg/kg) (Figure 4A). Further, as shown in Figures 4B–D, Western blot analysis revealed that the CCl_4_-induced p-Smad2/3 upregulation at the protein level was reversed in mice with CCl_4_-induced liver fibrosis following treatment with DOX (25 and 50 mg/kg).

### DOX inhibited HSC activation *in vitro*

**FIGURE 5:**
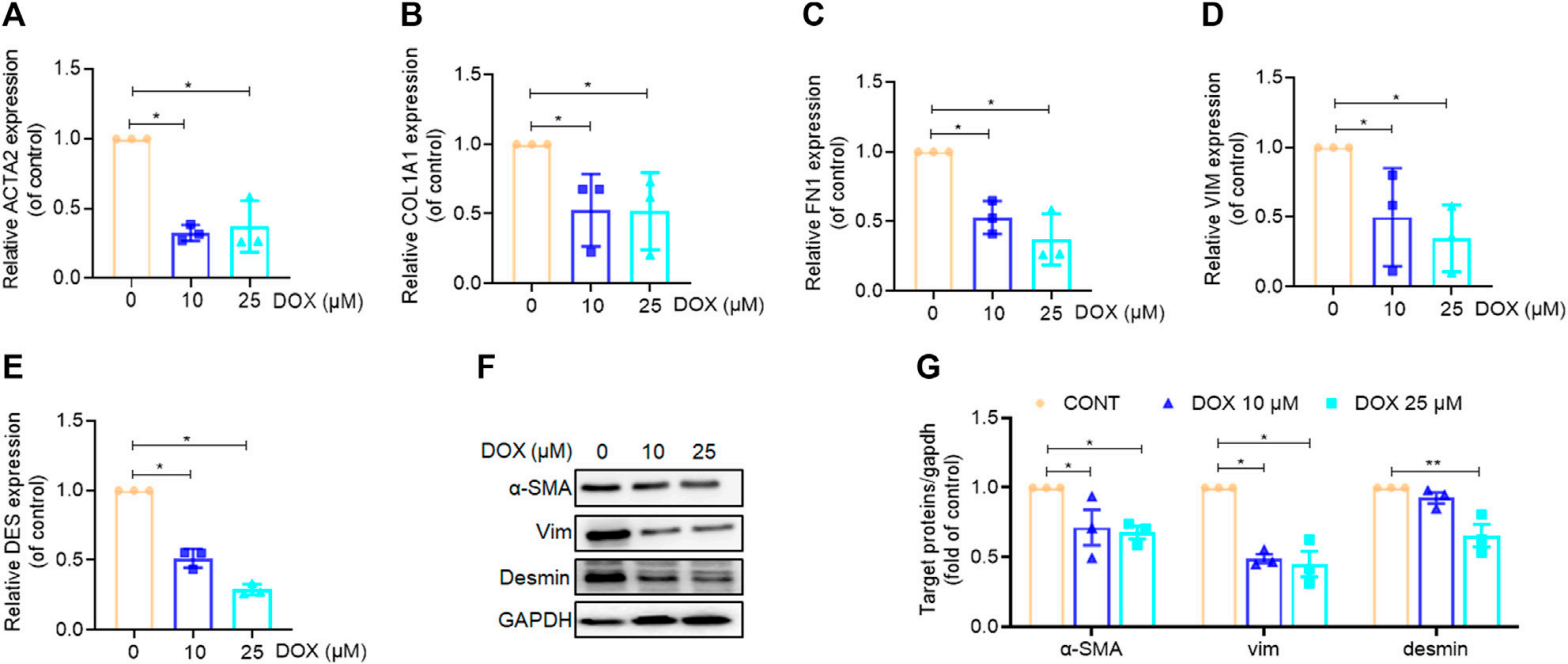
Effects of DOX inhibited HSC activation *in vitro*. LX-2 cells treated with DOX (10, 25 μM) for 48 h **(A–E)** LX-2 cell ACTA2 (α-SMA), COL1A1, FN1, VIM (Vimentin), DES (Desmin), mRNA expression (*n* = 3). **(F)** The expression of LX-2 cell α-SMA, vimentin and desmin proteins was detected by Western blot, and GAPDH was used as a loading control. **(G)** The quantitative result of α-SMA, vimentin and desmin. The results represent three independent experiments. Data were expressed as means ± SEM. *p < 0.05; **p < 0.01.

Our findings indicated that DOX ameliorated liver injury and fibrosis *in vivo*. Thus, we performed *in vitro* experiments to further corroborate these *in vivo* experimental results. Our findings, based on real-time PCR analysis, revealed that HSC activation markers, *α-*SMA, COL1A1, FN1, VIM, and DES were significantly downregulated by DOX in a dose-dependent manner (10 and 25 μM) following treatment for 48 h (Figures 5A–E). Moreover, western blot analysis revealed that DOX (10 and 25 μM) remarkably downregulated α-SMA, VIM, and desmin expression in activated HSCs (Figures 5F, G).

### DOX triggered activated HSC ferroptosis *in vitro*

**FIGURE 6:**
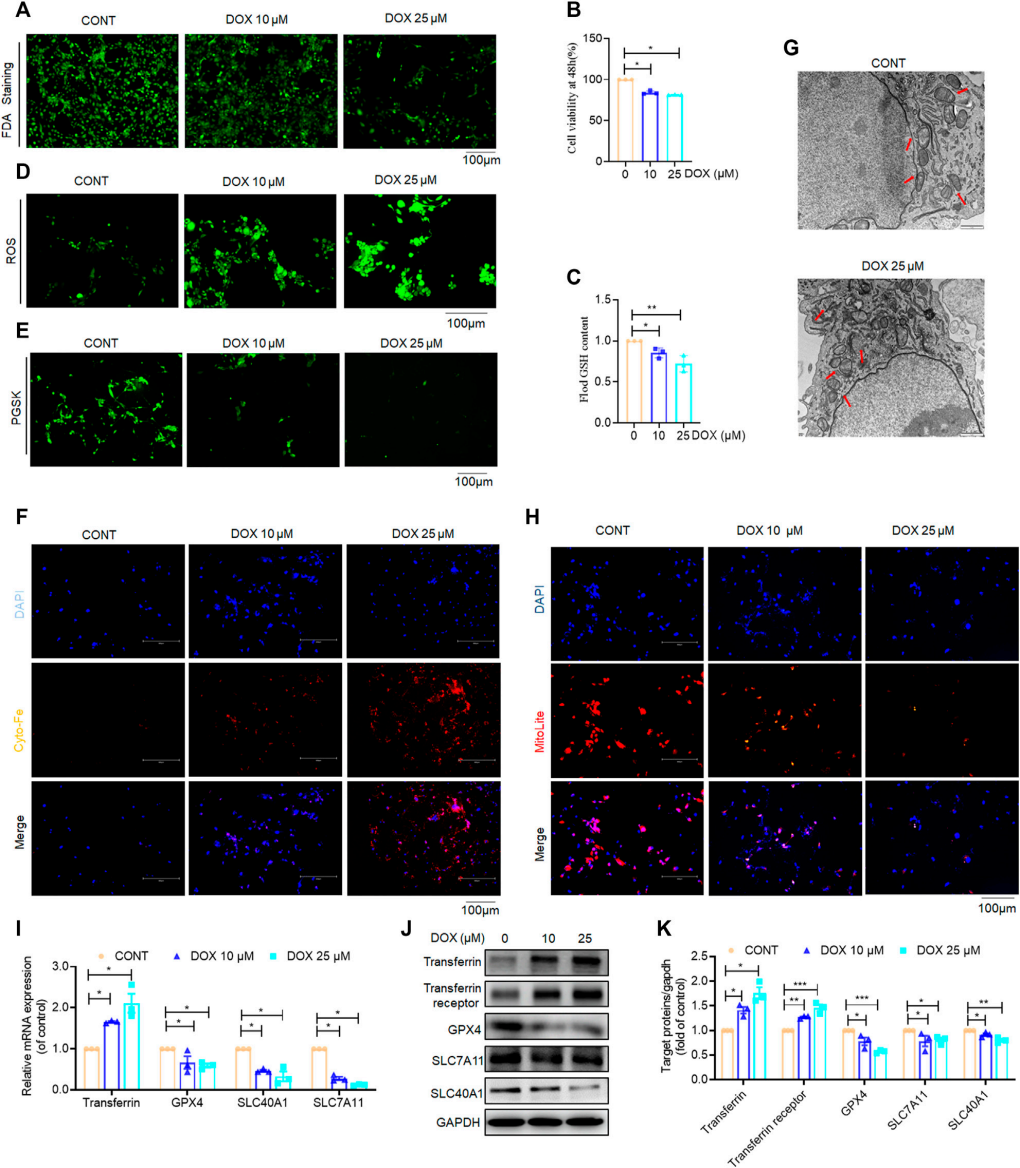
Effects of DOX triggers activated HSC ferroptosis *in vitro*. LX-2 was treated with DOX (10, 25 μM) for 48 h **(A)** FDA staining for evaluating cell death. LX-2 was stained in 10 μM FDA and the samples were observed with a fluorescence inverted microscope. **(B)** Cell Count Kit-8 analysis of cell viability in LX-2. CCK-8 was incubated with LX-2 at 37°C for 1 h and the samples was measured using a multifunctional enzyme labeler at 405 nm. **(C)** Cellular GSH amount (*n* = 3). **(D)** The ROS level in LX-2. LX-2 was stained in 10 μM DCFH-DA in DMEM for 1 h at 37°C and the samples were observed with a fluorescence inverted microscope. **(E)** Free iron levels in LX-2 were measured by Phen Green SK probe. LX-2 cells were stained in 10 μM Phen Green SK in PBS for 1 h at 37°C and the samples were imaged immediately. **(F)** Representative fluorescent images of intracellular iron level were identified by using FerroOrange (orange). Nuclei are stained by DAPI (blue). LX-2 was stained in 1 mM Ferro orange in PBS for 1 h at 37°C and constructs were imaged immediately. All samples as described **(E,F)** were observed with a fluorescence inverted microscope. **(G)** The mitochondria morphology was observed by transmission electron microscopy and mitochondrial length was summarized. **(H)** Mitochondrion staining detection. LX-2 cells were stained in the buffer containing a 1:500 dilution of MitoliteTM Red FX600 for 1 h at 37°C and the samples were observed with a fluorescence inverted microscope. **(I)** LX-2 cell transferrin, GPX-4, SLC7A11, SLC40A1 mRNA expression (*n* = 3). **(J)** The expression of LX-2 cell transferrin, transferrin receptor, GPX-4, SLC7A11, SLC40A1 proteins was detected by Western blot, and GAPDH was used as a loading control. **(K)** The quantitative result of transferrin, transferrin receptor, GPX-4, SLC7A11, SLC40A1. The results represent three independent experiments. For the statistics of each panel in this figure, data were expressed as means ± SEM. *p < 0.05; **p < 0.01; ***p < 0.001.

Previous *in vivo* and *in vitro* experiments have demonstrated that DOX significantly inhibits HSC activation; however, it is still unclear whether DOX treatment drives the ferroptosis of activated HSCs. Our findings in this study revealed that DOX induced HSC death at 48 h after treatment with different doses. Furthermore, FDA staining demonstrated reduced green fluorescence expression with increasing drug dosage, indicating that DOX significantly induced the death of activated HSCs (Figure 6A). A cell counting Kit-8 (CCK-8) analysis also showed that DOX remarkably suppressed HSC viability (Figure 6B). Reportedly, transferrin, GPX4, SLC7A11, and SLC40A1 are the key regulators of ferroptosis. Transferrin (TRF, Tf) is responsible for carrying iron absorbed by the digestive tract and released by the degradation of red blood cells (Gomme et al., 2005). The solute carrier family-7 member-11 (SLC7A11) is the main member of Systerm x c-, responsible for the uptake of extracellular cystine into the cytoplasm and the output of glutamic acid, which is then reduced to cysteine for the biosynthesis of GSH. GSH is synthesized from glutamic acid, cysteine and glycine, which directly affects the activity of GPX4 (Koppula et al., 2018). GPX4 is an anti-lipid peroxidase, responsible for catalyzing toxic unsaturated lipid hydroperoxides into non-toxic fatty alcohols, inhibiting ROS production, protecting cells from the effects of lipid peroxides, and playing an important role in inhibiting ferroptosis (Seibt et al., 2019). SLC40A1 (Solute Carrier Family 40 Member 1) is currently the only member of the SLC40 transporter family, and is currently the only transfer protein found to promote the transfer of iron from the cell to the outside of cell (Pietrangelo, 2004). We found that DOX (25, 50, 100, 250 μM) has no significant effect not only on the viability of liver parenchyma cells, but also on the expression of ferroptosis related proteins (Transferrin, GPX4, SLX7A11, SLC40A1, Supplementary Figure S1). Next, we investigated whether DOX mediated the activation of the ferroptotic mode *in vitro* by measuring ferroptosis-associated biomarkers (Hirschhorn and Stockwell, 2019). PGSK and FerroOrange were used for intracellular Fe^2+^ detection. As shown in Figures 6C–F, DOX treatment markedly triggered HSC ferroptosis, which was characterized by a decrease in GSH content, elimination of ROS scavenging enzymes, and increased iron levels, Since, we found differences which are statistically significant but slight, the biological meaning of these findings should be confirmed in further studies. Furthermore, electron microscopy images of mitochondria samples confirmed the occurrence of ferroptosis. We also observed that DOX-treated LX-2 cells showed smaller, ruptured, and broken mitochondria, which represent the morphological changes associated with ferroptotic cells, when compared with un-treated LX-2 cells (Cao and Dixon, 2016) (Figure 6G). Furthermore, the mitochondrial red fluorescent probe is a cationic dye that can selectively accumulate in the mitochondria through mitochondrial membrane potential gradients. The fluorescence intensity is proportional to the mitochondrial potential, and stronger fluorescence indicates higher mitochondrial potential, that is, healthier mitochondria, which proves that mitochondria are less affected by ferroptosis. In our results, mitochondrial staining revealed that DOX (10, 25 μM) significantly reduced the number of mitochondrion-positive HSCs (Figure 6H). Similarly, Our results show that DOX (10 and 25 μM) can increase the protein expression of transferrin and transferrin receptor in a dose-dependent manner; At the same time, in our study show that GPX4, SLC7A11, and SLC40A1 were reduced by DOX (10 and 25 μM) at both the mRNA and protein levels in LX-2 cells compared with the control (Figures 6I–K). Overall, these findings indicated that the inhibition of HSC activation is associated with DOX-induced HSC ferroptosis *in vitro*.

### Ferroptosis inhibition abolished DOX-induced anti-fibrosis effects *in vitro*

**FIGURE 7:**
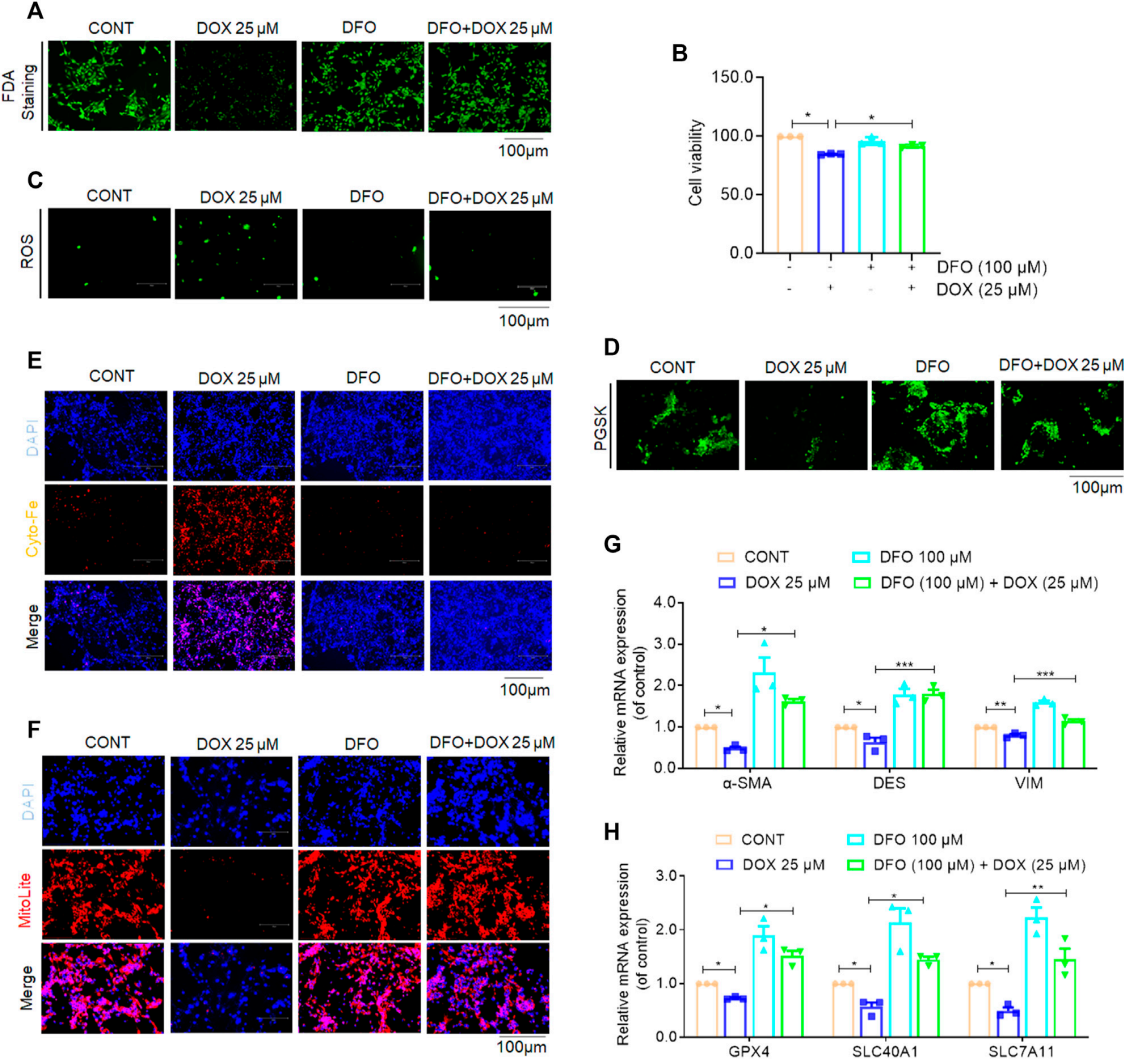
Inhibition of ferroptosis impaired DOX-induced anti-fibrosis effects *in vitro*. Activated LX-2 cells were exposed for 24 h to 100 μM DFO followed by 25 μM DOX treatment. **(A)** FDA staining for evaluating cell death. LX-2 was stained in 10 μM FDA and the samples were observed with a fluorescence inverted microscope. **(B)** Cell Count Kit-8 analysis of cell viability in LX-2. CCK-8 was incubated with LX-2 at 37°C for 1 h and the samples was measured using a multifunctional enzyme labeler at 405 nm. **(C)** The ROS level in LX-2. LX-2 was stained in 10 μM DCFH-DA in DMEM for 1 h at 37°C and the samples were observed with a fluorescence inverted microscope. **(D)** Free iron levels in LX-2 were measured by Phen Green SK probe. LX-2 cells were stained in 10 μM Phen Green SK in PBS for 1 h at 37°C and the samples were imaged immediately. **(E)** Representative fluorescent images of intracellular iron level were identified by using FerroOrange (orange). Nuclei are stained by DAPI (blue). LX-2 was stained in 1 mM Ferro orange in PBS for 1 h at 37°C and samples were imaged immediately. All samples as described **(D,E)** were observed with a fluorescence inverted microscope. **(F)** Mitochondrion staining detection. LX-2 cells were stained in the buffer containing a 1:500 dilution of MitoliteTM Red FX600 for 1 h at 37°C and the samples were observed with a fluorescence inverted microscope. **(G)** The mRNA expression of α-SMA, DES and VIM (*n* = 3). **(H)** The mRNA expression of GPX-4, SLC40A1 and SLC7A11 (*n* = 3). For the statistics of each panel in this figure, data were expressed as means ± SEM. *p < 0.05; **p < 0.01; ***p < 0.001.

To investigate whether ferroptosis plays a role in the anti-fibrotic effect of DOX, the ferroptosis-specific inhibitor, deferoxamine (DFO) was used to inhibit ferroptosis in activated HSCs (Wang et al., 2017). Additionally, activated LX-2 cells were exposed to DOX as well as DFO. The results of FDA staining as well as CCK-8 assay showed that LX-2 cells treated with DOX showed suppressed HSC viability, whereas DFO rescued this growth inhibition effect of DOX (Figures 7A, B). Additionally, DOX-treated LX-2 cells demonstrated remarkably increased ROS and iron levels compared with their untreated counterparts. Conversely, this effect on ferroptosis induction was weakened by co-treatment with DFO (Figures 7C–E). Mitochondrial staining further indicated that DOX treatment (25 μM) significantly reduced the number of mitochondria-positive cells, and DFO treatment significantly blocked the ferroptosis-promoting effect of DOX (Figure 7F). Real-time PCR analysis, performed to explore the expression of HSC activation- and ferroptosis-associated biomarkers showed that DOX treatment significantly downregulated the gene expression levels of the HSC activation- and ferroptosis-associated markers, whereas this function was inhibited by DFO (Figures 7G, H). Overall, these findings suggested that DFO inhibition abolished DOX-induced anti-fibrotic effects *in vitro*.

## Discussion

In this study, several key findings were made. First, we observed that DOX significantly attenuated hepatocellular injury and liver fibrosis in mice in the CCl_4_ group. Second, treatment with DOX significantly decreased the expression levels of HSC activation markers both *in vitro* and *in vivo*. Further, our results also indicated that DOX alleviated liver fibrosis by inducing ferroptosis in HSC and reducing ECM production. Thus, DOX may be useful in clinical practice as a treatment for liver fibrosis.

Liver fibrosis is a complex process, and its pathogenesis is based on increased synthesis or decreased degradation of the ECM, resulting in ECM deposition (Lee et al., 2015). HSCs normally store lipids in the liver. When the liver is damaged, they can be activated to become myofibroblasts, producing large amounts of ECM; therefore, their activation represents the pathogenesis of liver fibrosis (Tacke and Trautwein, 2015). This also implies that inducing activated HSC death is an important strategy for the treatment of liver fibrosis. Ferroptosis, a newly discovered form of programmed cell death, is significantly different from other types of programmed death processes, such as apoptosis, necrosis, and autophagy, at the morphological, biochemical, and genetic levels (Dixon et al., 2012). Several recent studies indicate that a variety of cytokines, signaling pathways (Zhang et al., 2020a; Zhang et al., 2020b; Z; Zhang et al., 2018; Zhu et al., 2021) affect the occurrence of ferroptosis and participate in liver fibrosis development. Tripartite motif-containing protein 26 (TRIM26) induces HSC ferroptosis through the regulation of solute carrier family-7 member-11 (SLC7A11) ubiquitination, thereby ameliorating liver fibrosis (Zhu et al., 2021). In addition to the above-mentioned targets for regulating ferroptosis, it has also been reported that several drugs can treat liver fibrosis by inducing ferroptosis in HSCs. Specifically, sorafenib and erastin can induce ferroptosis in HSCs to alleviate liver fibrosis (Z. Zhang et al., 2018). Magnesium isoglycyrrhizinate (MgIG) can upregulate heme oxygenase 1 (HO-1) expression, leading to intracellular iron deposition, lipid peroxide accumulation, the induction of ferroptosis in HSC, and the inhibition of liver fibrosis (Sui et al., 2018). Further, artesunate ultimately induces ferroptosis in HSCs and ameliorates liver fibrosis development by depleting glutathione (GSH), reducing glutathione peroxidase 4 (GPX4) activity, and favoring ROS accumulation (Kong et al., 2019). Interestingly, in this current study we also identified that a drug with high efficacy and low toxicity, DOX, alleviated liver fibrosis and induced ferroptosis in HSCs.

DOX has been used for patients with asthma and chronic obstructive pulmonary disease (Page, 2010). DOX increases intracellular cyclic adenosine monophosphate concentrations by inhibiting intracellular phosphodiesterase activity, and this in turn reduces the incidence of airway pressure and barometric injury in patients (Y. Zhang et al., 2016), and reportedly, DOX, *via* nucleotide-binding oligomerization domain, leucine-rich repeat and pyrin domain-containing 3-thioredoxin-interacting protein (NLRP3-TXNIP) inflammasome activation, mitigates epithelial inflammation (Jiao et al., 2020). Moreover, compared with aminophylline, DOX has fewer cardiovascular adverse effects, a higher safety profile, and better user experience (Rao et al., 2013). Based on these previous studies, we investigated whether DOX exerts a therapeutic effect on liver fibrosis as well as the possible mechanisms.

Studies have shown that hepatocyte injury is one of the key factors to promote the formation of liver fibrous scar (Kisseleva and Brenner, 2021). In our study showed that DOX also reduced the elevated serum ALT/AST activities in CCl_4_-treated mice, and the liver histological analysis showed that DOX significant reduce hepatic infiltration of immune cells, swelling and necrosis of hepatocytes in CCl_4_-treated mice. These evidences show that DOX protection against hepatocellular injury induced by CCl_4_. We observed that DOX ameliorated liver fibrosis and inhibited HSC activation both *in vivo* and *in vitro*. Additionally, our findings indicated that DOX treatment significantly impaired HSC viability *via* inducing ferroptosis. Primarily, Electron microscopy and mitochondrial staining provided direct evidence that DOX induced ferroptosis in HSCs. Furthermore, the ferroptosis markers iron and ROS level in HSCs increasing significantly induced by DOX, while GSH content was decreased. Meanwhile, DOX can increase the protein expression of transferrin and transferrin receptor in a dose-dependent manner; GPX4, SLC7A11, and SLC40A1, which have been recognized as critical upstream factors of ferroptosis (Pietrangelo, 2004; Gomme et al., 2005; Koppula et al., 2018; Seibt et al., 2019), was inhibited significantly by DOX. Then the inhibition of ferroptosis using the specific inhibitor, DFO not only abolished the ferroptosis-inducing effect of DOX, but also resisted its anti-fibrosis effect. These findings above indicated that the anti-fibrotic effect of DOX is closely related to the induction of ferroptosis in HSCs.

**FIGURE 8:**
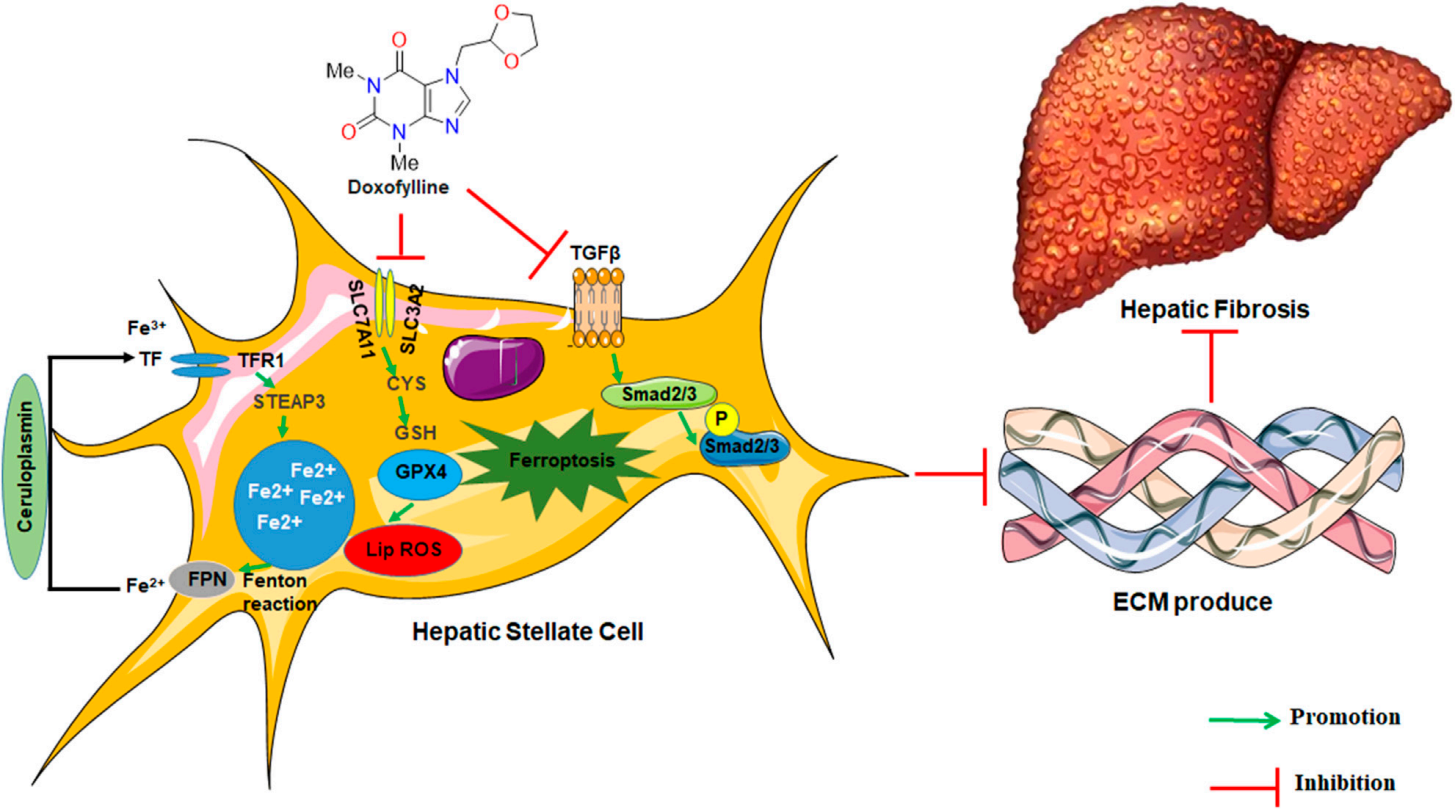
The graphic illustration of the mechanism of doxofylline protecting against liver fibrosis.

In conclusion, our findings overall indicated for the first time that DOX elicits its anti-fibrotic effects through the regulation of HSC ferroptosis (Figure 8). Additionally, DOX-only treatment of mice without liver fibrosis exhibited no significant effect on serum ALT/AST levels or liver histomorphology. This observation suggests that DOX may be useful in clinical settings as treatment of liver fibrosis. However, in this study, we did not explore the *in vivo* distribution of DOX; this was a limitation of this study. Therefore, the further evaluation of the *in vivo* metabolic profile of DOX should be realized in future studies.

## Data Availability

The original contributions presented in the study are included in the article/Supplementary Material, further inquiries can be directed to the corresponding authors.

## Glossary

ALT/AST: Alanine/Aspartate aminotransferase
ANOVA: Analysis of variance
α-SMA: α-Smooth muscle actin
BSA: Bovine serum albumin
CCK-8: Cell counting Kit-8
CCl4: Carbon tetrachloride
cDNA: Complementary deoxyribonucleic acid
DDR2: Discoidin domain receptor 2
DAB,3: 3-Diaminobenzidine tetrahydrochloride
DAPI: 4′, 6-diamidino-2-phenylindole
DCFH-DA: Dichlorodihydrofluorescein diacetate
DES: Desmin
DFO: deferoxamine
DMEM: Dulbecco’s modified eagle medium
DOX: Doxorubicin
ECM: Extracellular matrix
FBS: Fetal bovine serum
FDA: Fluorescein diacetate
FITC: Fluorescein isothiocyanate
GAPDH: Glyceraldehyde-3-phosphate dehydrogenase
GPX4: Glutathione peroxidase 4
GSH: glutathione
H&E: Hematoxylin-eosin
HO-1: Heme oxygenase 1
NLRP3-TXNIP: Nucleotide-binding oligomerization domain, leucine—rich repeat and pyrin domain-containing 3—thioredoxin-interacting protein
HSC: Hepatic stellate cell
LSD: Least significant difference
MMP-2: Matrix metalloproteinase
MgIG: Magnesium isoglycyrrhizinate
Ob-RL: Obese receptor long form
PDGF-R: Platelet derived growth factor receptor β
PUFA: Peroxidizing polyunsaturated fatty acid
PBS: Phosphate buffer saline
PDE: Phosphodiesterase
PGSK: Phen Green SK diacetate
RNA: ribonucleic acid
ROS: Reactive oxygen species
SEM: Standard error of the mean
SIL: Silymarin
SLC7A11: Solute carrier family-7 member-11
SLC40A1: Solute carrier family 40 member 1
Smad 2/3: SMAD family member 2/3
TIMP-2: Matrix metalloproteinase-2
TEM: Transmission electron microscope
TGF-β: Transforming growth factor-beta
VIM: Vimentin
