# Supplementary material for: Doxofylline ameliorates liver fibrosis by regulating the ferroptosis signaling pathway
Source: Front Pharmacol. 2023 Mar 17;14:1135366. doi: 10.3389/fphar.2023.1135366 (PMC10063813; doi:10.3389/fphar.2023.1135366)
Supplement: Supplementary file 1 [file DataSheet1.docx]

***Supplementary Materials and Methods***

**Doxofylline ameliorates liver fibrosis by regulating the ferroptosis signaling pathway**

**Supplementary Table S1: Primer sequences for qPCR.**

| Target | Full gene name | Gene ID | Primer | Sequence(5'-3') |
| --- | --- | --- | --- | --- |
| Col1a1 (mouse) | collagen, type I, alpha 1 | 12842 | FP | TGACTGGAAGAGCGGAGAGT |
|  |  |  | RP | GACGGCTGAGTAGGGAACAC |
| Tgfb1 (mouse) | Transforming growth factor, beta 1 | 21803 | FP | TGCCCTCTACAACCAACACA |
|  |  |  | RP | GTTGGACAACTGCTCCACCT |
| Acta2 (mouse) | actin, alpha 2, smooth muscle, aorta | 11475 | FP | GGGAGTAATGGTTGGAATGG |
|  |  |  | RP | GGTGATGATGCCGTGTTCTA |
| Col3a1  (mouse) | collagen, type III, alpha 1 | 12825 | FP | CTGGCCCTCCTGGTGCTTCT |
|  |  |  | RP | CCTTGGCCCATCCTTTCCTG |
| Vim  (mouse) | vimentin | 22352 | FP | CGGAAAGTGGAATCCTTGCAGG |
|  |  |  | RP | AGCAGTGAGGTCAGGCTTGGAA |
| Des  (mouse) | desmin | 13346 | FP | GCGGCTAAGAACATCTCTGAGG |
|  |  |  | RP | ATCTCGCAGGTGTAGGACTGGA |
| Actb (mouse) | actin, beta | 11461 | FP | TTCGTTGCCGGTCCACACCC |
|  |  |  | RP | GCTTTGCACATGCCGGAGCC |
| ACTA2  (human) | actin alpha 2, smooth muscle | 59 | FP | CTATGCCTCTGGACGCACAACT |
|  |  |  | RP | CAGATCCAGACGCATGATGGCA |
| VIM  (human) | vimentin | 7431 | FP | AGGCAAAGCAGGAGTCCACTGA |
|  |  |  | RP | ATCTGGCGTTCCAGGGACTCAT |
| DES  (human) | desmin | 1674 | FP | TCCAGTCCTACACCTGCGAGAT |
|  |  |  | RP | CGCAATGTTGTCCTGGTAGCCA |
| FN1  (human) | fibronectin 1 | 2335 | FP | ACAACACCGAGGTGACTGAGAC |
|  |  |  | RP | GGACACAACGATGCTTCCTGAG |
| COL1A1  (human) | collagen type I alpha 1 chain | 1277 | FP | GATTCCCTGGACCTAAAGGTGC |
|  |  |  | RP | AGCCTCTCCATCTTTGCCAGCA |
| GPX4  (human) | glutathione peroxidase 4 | 2879 | FP | ACAAGAACGGCTGCGTGGTGAA |
|  |  |  | RP | GCCACACACTTGTGGAGCTAGA |
| SLC7A11  (human) | solute carrier family 7 member 11 | 23657 | FP | TCCTGCTTTGGCTCCATGAACG |
|  |  |  | RP | AGAGGAGTGTGCTTGCGGACAT |
| SLC40A1(human) | solute carrier family 40 member 1 | 30061 | FP | GAGACAAGTCCTGAATCTGTGCC |
|  |  |  | RP | TTCTTGCAGCAACTGTGTCACAG |
| ACTB  (human) | actin beta | 60 | FP | CACCATTGGCAATGAGCGGTTC |
|  |  |  | RP | AGGTCTTTGCGGATGTCCACGT |

FP, Forward Primer; RP, Reverse Primer


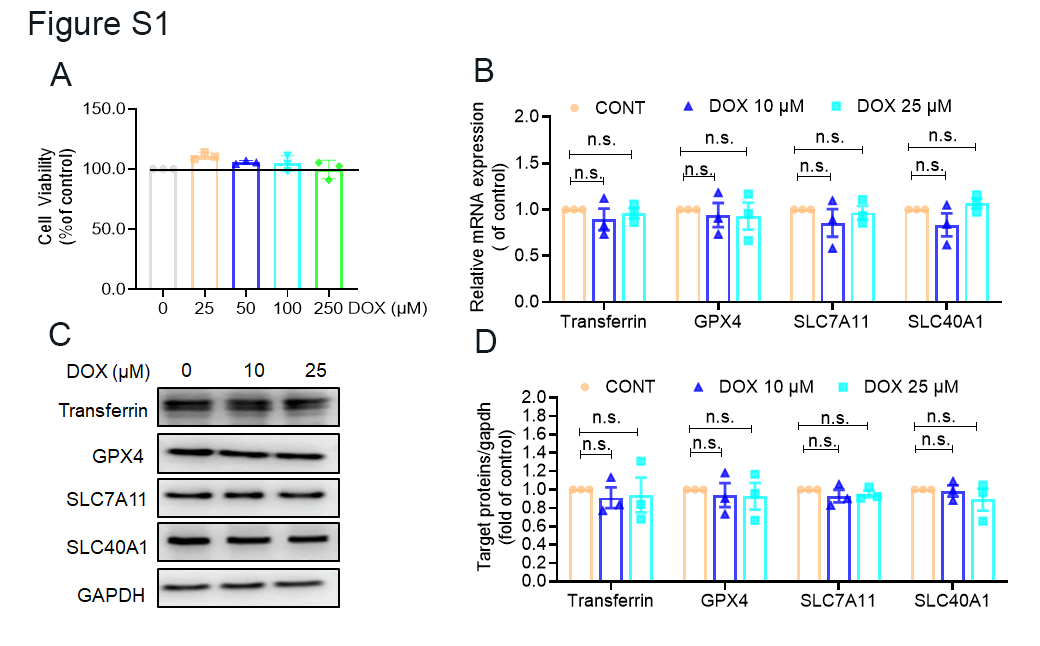


**Supplementary Figure S1**

Effect of DOX on ferroptosis related indexes of hepatic parenchymal cells heparg. (A) Heparg were treated with DOX (25, 50, 100, 250 μM) for 48 h. Cell Count Kit-8 analysis of cell viability in Heparg. (B) Heparg cell Transferrin, GPX-4, SLC7A11, SLC40A1 mRNA expression (n=3). (C) The expression of Heparg cell Transferrin, GPX-4, SLC7A11, SLC40A1 proteins was detected by western-blot, and GAPDH was used as a loading control. (D) The quantitative result of Transferrin, GPX-4, SLC7A11, SLC40A1. The results represent three independent experiments. For the statistics of each panel in this figure, data were expressed as means ± SEM (n=3).
